# Do New World pitvipers “scale‐down” at high elevations? Macroecological patterns of scale characters and body size

**DOI:** 10.1002/ece3.5486

**Published:** 2019-07-31

**Authors:** Robert C. Jadin, Joseph R. Mihaljevic, Sarah A. Orlofske

**Affiliations:** ^1^ Department of Biology University of Wisconsin Eau Claire Eau Claire WI USA; ^2^ School of Informatics, Computing, and Cyber Systems Northern Arizona University Flagstaff AZ USA; ^3^ Department of Biology University of Wisconsin Stevens Point Stevens Point WI USA

**Keywords:** Bergmann's rule, biogeography, body size, Crotalinae, macroecology, Viperidae

## Abstract

Bergmann's rule describes the macroecological pattern of increasing body size in response to higher latitudes and elevations. This pattern is extensively documented in endothermic vertebrates, within and among species; however, studies involving ectotherms are less common and suggest no consistent pattern for amphibians and reptiles. Moreover, adaptive traits, such as epidermal features like scales, have not been widely examined in conjunction with Bergmann's rule, even though these traits affect physiological processes, such as thermoregulation, which are hypothesized as underlying mechanisms for the pattern. Here, we investigate how scale characters correlate with elevation among 122 New World pitviper species, representing 15 genera. We found a contra‐Bergmann's pattern, where body size is smaller at higher elevations. This pattern was mainly driven by the presence of small‐bodied clades at high elevations and large‐bodied clades at low elevations, emphasizing the importance of taxonomic scope in studying macroecological patterns. Within a subset of speciose clades, we found that only *Crotalus* demonstrated a significant negative relationship between body size and elevation, perhaps because of its wide elevational range. In addition, we found a positive correlation between scale counts and body size but no independent effect of elevation on scale numbers. Our study increases our knowledge of Bergmann's rule in reptiles by specifically examining characters of squamation and suggests a need to reexamine macroecological patterns for this group.

## 1 INTRODUCTION

One of the enduring challenges in macroecology and environmental biology is characterizing the variation in life history traits along environmental gradients and understanding potential underlying mechanisms (Brown, 1995; Gaston & Blackburn, 2000; Olalla‐Tárraga, Rodríguez, & Hawkins, 2006; Pincheira‐Donoso, Hodgson, & Tregenza, 2008; Terribile, Olalla‐Tárraga, Diniz‐Filho, & Rodríguez, 2009). Among the “ecogeographical rules” describing spatial patterns in biological traits, correlations of body size with geographic or climatic characteristics are the most broadly investigated (Ashton, Burke, & Layne, 2007; Gaston, Chown, & Evans, 2008; Moreno Azócar et al., 2015). Known as Bergmann's rule, it specifically refers to the increase in animal body size among closely related species with decreasing temperature (Bergmann, 1847; Blackburn, Gaston, & Loder, 1999; Gaston et al., 2008; Moreno Azócar et al., 2015). This pattern is considered an evolutionary response to latitudinal gradients (Bergmann, 1847), which are similar to elevation gradients in the physiological and life history constraints imposed by changes in temperature, precipitation, and other correlated environmental variables (Ashton & Feldman, 2003; Blackburn & Ruggiero, 2001; Cruz, Fitzgerald, Espinoza, & Schulte, 2005; Gaston et al., 2008).

Controversy over the study of Bergmann's rule has stemmed from different interpretations of taxonomic scales and the importance of incorporating mechanisms (Terribile et al., 2009; Watt, Mitchell, & Salewski, 2010). Previous work has investigated patterns within and among species, genera, orders, and class, with the requirement of monophyly of the examined taxa (Adams & Church, 2008; Cruz et al., 2005; Gaston et al., 2008; Olalla‐Tárraga et al., 2006; Watt et al., 2010). However, empirical studies examining intraspecific patterns (i.e., James' rule) outnumber interspecific studies (i.e., Bergmann's rule; Gaston et al., 2008; Olalla‐Tárraga et al., 2006). Likewise, numerous mechanisms have been proposed for Bergmann's rule, which generally fit into the categories of heat conservation and balance, resource availability (i.e., fasting endurance, seasonality, primary productivity, starvation resistance, energy budgets), and competition (Ashton & Feldman, 2003; Adams & Church, 2008; Moreno Azócar et al., 2015; Olalla‐Tárraga et al., 2006; Pincheira‐Donoso & Meiri, 2013; Reed, 2003; Terribile et al., 2009; Watt et al., 2010). The original rule pertained to the surface area to volume ratio of endotherms because larger body sizes increase heat conservation (Moreno Azócar et al., 2015; Watt et al., 2010). There are many fewer studies on Bergmann's rule in ectothermic taxa, likely due to disagreements over which mechanisms (if any) might apply to this group (Moreno Azócar et al., 2015; Olalla‐Tárraga, 2011; Pincheira‐Donoso et al., 2008; Terribile et al., 2009; Watt et al., 2010).

Independent of specific morphological or physiological mechanisms, ectotherm physiology is strongly related to body temperature, which influences metabolism, and therefore affects survival and reproduction (Pincheira‐Donoso et al., 2008; Pincheira‐Donoso & Meiri, 2013). But, compared with endotherms, ectotherms exhibit different capacities to deal with heat imbalance. For example, some are thermoregulators (i.e., animals with good thermoregulating abilities) while others are thermoconformers (i.e., animals with body temperatures fluctuating more closely to ambient temperature; Olalla‐Tárraga & Rodríguez, 2007). In terrestrial ectotherms, behavioral and physiological mechanisms are potentially important for heat regulation in addition to the effect of body mass (Stevenson, 1985). Based on these differences, it may not be surprising that existing studies on the relationship between body size and elevation in ectotherms have not only found typical Bergmann patterns (amphibians, Olalla‐Tárraga & Rodríguez, 2007; lizards, Cruz et al., 2005; reviewed in Watt et al., 2010) but also contra‐Bergmann patterns (i.e., a negative relationship between body size and temperature, or a proxy for temperature; Cowles, 1945; salamanders, Olalla‐Tárraga & Rodríguez, 2007), or no detectable patterns (e.g., plethodontid salamanders, Feder, Papenfuss, & Wake, 1982, Adams & Church, 2008; lizards, Pincheira‐Donoso et al., 2008). Among ectotherms, broad empirical support for the pattern across reptiles is lacking owing in part to relatively few studies (Adams & Church, 2008; Olalla‐Tárraga et al., 2006; Reed, 2003).

Squamate reptiles (i.e., lizards and snakes) do not exhibit consistent patterns of body size along environmental gradients, and a single mechanism may not explain this variability (Olalla‐Tárraga et al., 2006; Watt et al., 2010). Beginning with one of the earliest macroecological studies on lizards, Bogert (1949) found that larger species inhabited warm, low elevation areas, while smaller species were found in cooler, higher elevations (i.e., a contra‐Bergmann pattern), which was recently observed in *Sceloporus* (Oufiero, Gartner, Adolp, & Garland, 2011). Across several families of snakes, Lindsey (1966) observed a slight tendency of larger species inhabiting regions with lower temperatures. Later, Reed (2003) found little support for Bergmann's rule in either Elapidae or Viperidae. Finally, a review by Millien et al. (2006) highlighted that snakes were the vertebrate group with the lowest agreement with Bergmann's rule. This deviation from the normal pattern of Bergmann's rule among snakes might be due to their elongated bodies that impact heat exchange through relatively high surface area to volume ratios (Feldman & Meiri, 2014; Lillywhite, 1987; Lindsey, 1966; Olalla‐Tárraga & Rodríguez, 2007).

Squamates in particular possess keratinized scales that may play important roles in water balance presumably driving strong correlations between elevation and scale numbers, such as patterns found in *Sceloporus* (Acevedo, 2009). Variation in climate could select for differences in the size, shape, number, color, and perhaps other features of scales. For example, large scales are related to latitude (Bogert, 1949; Oufiero et al., 2011) but also to larger species of lizards. In addition, scale number can vary intraspecifically along altitudinal gradients (e.g., *Anolis marmoratus*, Malhotra & Thrope, 1994). Other environmental factors like precipitation may also influence scale characters. In lizards, larger and fewer scales are observed in hot, dry regions (Hellmich, 1951; Horton, 1972; Lister, 1976; Sanders, Malhotra, & Thorpe, 2004), while snakes have more scales in these types of habitats (Brown, Thorpe, & Baez, 1991; Klauber, 1941, 1997; Licht & Bennett, 1972; Malhotra & Thrope, 1994; Soulé & Kerfoot, 1972; Thorpe & Báez, 1987). One hypothesis is that heat and water balance is related to the amount of exposed interstitial skin, which is influenced by scale size or number (Pough et al., 2001; Sanders et al., 2004). Alternatively, environmental conditions such as temperature during development may influence scale characters (Osgood, 1978). Body size is also often positively correlated with the number of ventral scales (Klauber, 1945; Lindell, Forsman, & Merila, 1993) while the distribution of body size has also been shown to vary along environmental gradients (Bogert, 1949; Pincheira‐Donoso et al., 2008). Additional studies are needed to understand the range of patterns and the complex interplay between environmental gradients, body size, and scale characteristics in squamate reptiles in order to test potential mechanisms.

An excellent model to study macroecological patterns in reptiles is New World pitvipers, an ecologically and morphologically diverse, broadly distributed group with limited dispersal for a vertebrate. As carnivores, studying these snakes also reduces the influence of varying trophic level (Reed, 2003). Furthermore, the lineage is monophyletic, meeting a key requirement of Bergmann's rule (Cruz et al., 2005; Reed, 2003) and has a well‐resolved phylogeny supporting comparative analyses among clades (Castoe & Parkinson, 2006; Gutberlet & Harvey, 2004; Jadin, Smith, & Campbell, 2011; Kraus, Mink, & Brown, 1996). In this study, we use information on evolutionary relationships and previously published data on geographic distribution and morphological traits to assess correlations between scale counts, body size, and elevation across lineages of New World pitvipers. We investigate whether a pattern emerges that suggests environmental change across elevation gradients produces physiological constraints and, therefore, selects for body size and scale characters. We predict that increasing elevation will be associated with smaller body size. This extends previous work investigating snake body size and elevation while also investigating important morphological traits (Reed, 2003; Terribile et al., 2009).

## 2 METHODS

### 2.1 Study system

**Figure 1:**
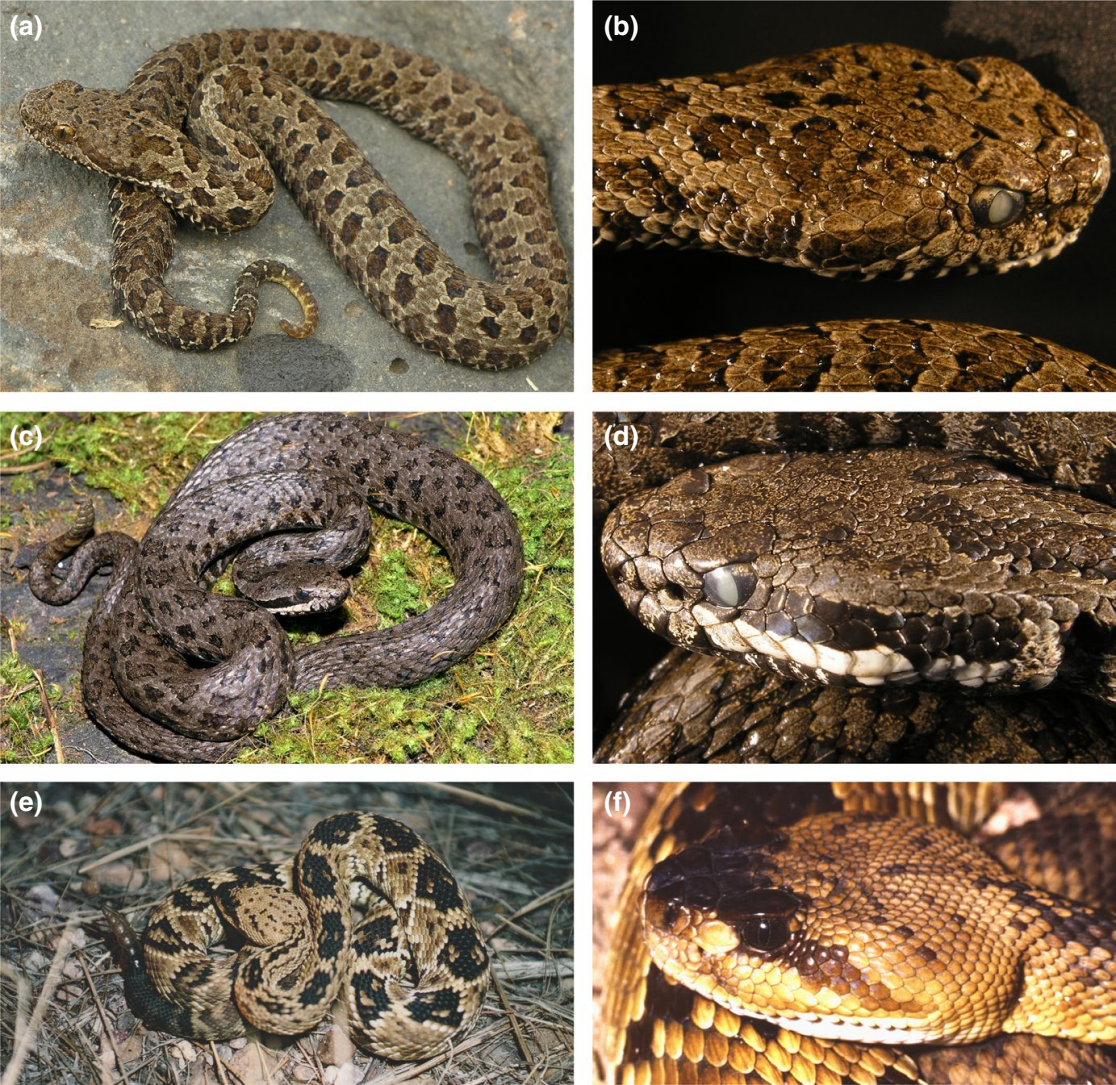
Two distantly related species, *Mixcoatlus browni* (a, b) and *Crotalus intermedius* (c, d), showing similar body size (maximum TL equals 51.5 and 57 centimeters, respectively), color pattern, and scale numbers (e.g., few scales on the head). This contrasts the distinction between *C. intermedius* (c, d) and its closer relative *C. molossus* (e, f), which lives in lowland, arid areas and has a larger body size (max TL 133 cm), different color pattern, and a greater number of scales. Photographs were taken by RCJ (b, d, e), Eric N. Smith (a, c), and Jonathan A. Campbell (f)

In the New World, pitvipers range in distribution from Canada (e.g., Prairie Rattlesnake, *Crotalus viridis*) to southern Argentina (Patagonian Lanceheads, *Bothrops ammodytoides*) and from the Pacific coast (e.g., Northern Pacific Rattlesnake, *Crotalus oreganus*) to the Atlantic coast (e.g., *Bothrops leucurus*). They are found from elevations above 3,000 meters (e.g., Barbour's Mexican Montane Pitviper, *Mixcoatlus barbouri*; Figure 1a,b) to sea level (e.g., Eastern Diamondback Rattlesnake, *Crotalus adamanteus*) and from desert habitats (e.g., Sidewinder, *Crotalus cerastes*) to lowland rainforest (e.g., Speckled Forest Pitviper, *Bothriopsis taeniata*). Some of these species have extensive ranges (e.g., Terciopelo, *Bothrops asper*) while others are geographically isolated (e.g., Golden Lancehead, *Bothropoides insularis*). Most species of New World pitvipers are terrestrial, but some species are arboreal (e.g., Mexican Horned Pitviper, *Ophryacus undulatus*) or even semiaquatic (e.g., Cottonmouth, *Agkistrodon piscivorus*).

In addition to their broad geographic and ecological distribution, New World pitvipers have extensive morphological diversity (see Figure 1). They range in size from adults of little more than 50 cm in length (e.g., Tzotzil Middle American Montane Pitviper, *Cerrophidion tzotzilorum*) to records exceeding 3.5 m in length (South American Bushmaster, *Lachesis muta*). Among these species, the range of scale numbers is considerable from mid‐dorsal scale rows (17–37) to ventrals (103–254) showing considerable variation that can be selected upon throughout their extensive distributions. This makes New World pitvipers an ideal clade to examine patterns of biodiversity and biogeography across North, Central, and South America.

### 2.2 Clade assessment

Essential to understanding the patterns of species distributions along environmental gradients requires incorporation of phylogenetic information in data analysis (Gaston et al., 2008; Harvey & Pagel, 1991). Importantly, our understanding of the diversity and evolutionary relationships of pitvipers has become quite robust over the past two decades with a strong congruence between gross morphology and mitochondrial genes (see review in Gutberlet & Harvey, 2004). Phylogenetic relationships within most of the New World pitviper genera are strongly supported, and individual species appear to be accurately assigned to their respective genera. For example, there is strong support for relationships within the *Porthidium* group (genera *Atropoides*, *Cerrophidion*, and *Porthidium*; Castoe, Sasa, & Parkinson, 2005; Jadin, Gutberlet, & Smith, 2010; Jadin, Townsend, Castoe, & Campbell, 2012), the rattlesnakes (genera *Crotalus* and *Sistrurus*; Reyes‐Velasco, Meik, Smith, & Castoe, 2013; Blair & Sánchez‐Ramírez, 2016), the South American lanceheads (genera *Bothriopsis*, *Bothrocophias*, *Bothropoides*, *Bothrops*, and *Rhinocerophis*; Fenwick, Gutberlet, Evans, & Parkinson, 2009 but see Carrasco, Mattoni, Leynaud, & Scrocchi, 2012), and the Mexican highland endemic pitvipers (genera *Mixcoatlus* and *Ophryacus*; Jadin et al., 2011). Nevertheless, morphological and molecular datasets do not agree or show strong support for many of the relationships among these clades, and singular genera such as *Agkistrodon* (Cantils, Copperheads, and Cottonmouths), *Bothriechis* (Palm‐pitvipers), or *Lachesis* (Bushmasters). This lack of phylogenetic resolution concerning how the genera are related constitutes a large knowledge gap that hinders phylogenetic comparative analyses. Therefore, we grouped the species of New World pitvipers within their respective genera as well as some strongly supported clades composed of several closely related genera (e.g., *Porthidium* group) for statistical analyses. We conducted our analysis on several of these smaller clades and within particular genera to examine whether or not patterns were apparent across different taxonomic scales (Meiri & Thomas, 2007).

### 2.3 Body size, scale counts, and elevation data

We used the literature to obtain data on scale morphology, body size, and elevation for 122 of the 150 currently described species of New World pitvipers (Uetz, 2019). These data were generally taken from Campbell and Lamar (2004) but data from several recently described or revised taxa were obtained from other published sources (Table 1). For each species, we derived a single value for each scale character of interest, obtained from data within the geographic range of the taxa (Gaston et al., 2008). This method is unaffected by species richness and is conservative by only considering each species once as opposed to grid‐based methods (Meiri & Thomas, 2007). Specifically, we used the maximum total length recorded as our measurement of body size as this is recommended for use in snakes because of their slender, elongated bodies, and average body size is unavailable in the literature (Terribile et al., 2009). For squamation, we recorded number of intersupraoculars, mid‐dorsal scale rows, subcaudals, supralabials, and ventrals (as described in Klauber, 1997). Most of these values were found with a range of values (e.g., ventrals), and we therefore recorded the mode (as provided in Campbell & Lamar, 2004) or calculated a mean, rounding up in cases of nonintegers. Elevation for species occurrence was obtained from the literature, and we calculated a midpoint between the range given for the normal elevation distributions for each species (Blackburn & Hawkins, 2004; Gaston, 2003; Olalla‐Tárraga, Bini, Diniz‐Filho, & Rodríguez, 2010). Additional analyses using both lowest and highest elevation values were conducted to ensure that the choice of elevation metric did not influence the results of the study.

**Table 1:** Data of elevation, scalation, and maximum body length (in centimeters) of New World pitvipers

Species	Elev	MDSR	SL	Ven	SC	ISO	TL
*Agkistrodon bilineatus*	300	23	8	136	59	1	138
*Agkistrodon contortrix*	500	23	8	148	50	1	137.2
*Agkistrodon piscivorus*	300	25	8	137	50	1	180
*Agkistrodon taylori*		23	8	133	48	1	96
*Atropoides indomitus* a	935	24	11	138	34	11	65.8
*Atropoides mexicanus*	820	25	10	125	31	8	97.9
*Atropoides nummifer* b	1,135	25	10	131	33	9	69.5
*Atropoides occiduus*	1,300	25	9	131	30	9	79.5
*Atropoides olmec*	1,015	23	11	111	29	10	77
*Atropoides picadoi*	1,150	25	9	147	35	9	120.2
*Bothriechis aurifer*	1,750	19	10	158	57	3	100
*Bothriechis bicolor*	1,250	21	10	166	66	10	100
*Bothriechis guifarroi* c	1,233	19	10	164	64	5	76.5
*Bothriechis lateralis*	1,492	23	10	163	62	7	100
*Bothriechis marchi*	1,000	19	11	165	62	5	104
*Bothriechis nigroviridis*	2,075	19	10	146	51	7	93.7
*Bothriechis rowleyi*	1,445	19	10	160	60	4	97.3
*Bothriechis schlegelii*	650	23	9	153	53	8	97.9
*Bothriechis supraciliaris*	1,250	23	9	146	50	8	80
*Bothriechis thalassinus* d	1,308	21	11	165	64	7	100
*Bothriopsis bilineata*	500	27	7	205	66	7	123
*Bothriopsis chloromelas*	1,500	24	8	186	52	6	100
*Bothriopsis medusa*	1,238	21	7	161	54	4	80
*Bothriopsis oligolepis*	2,000	23	8	192	60	7	98.6
*Bothriopsis pulchra*	1,650	21	7	174	64	7	76.4
*Bothriopsis taeniata*	1,067	27	7	229	79	7	175
*Bothrocophias andianus*	2,550	22	7	168	56	7	125.8
*Bothrocophias campbelli*	1,650	23	8	165	56	6	123
*Bothrocophias colombianus*	1,300	25	9	168	53	8	136
*Bothrocophias hyoprora*	500	23	8	131	48	6	83
*Bothrocophias microphthalmus*	1,675	23	8	153	51	6	116.2
*Bothrocophias myersi*	138	23	7	145	48	5	75.6
*Bothrocophias pictus*	1,400	23	10	166	38	6	60
*Bothropoides alcatraz*	135	25	9	180	50	7	50.5
*Bothropoides diporus*	350	25	9	178	49	8	110
*Bothropoides erythromelas*	1,000	20	8	149	37	6	85
*Bothropoides insularis*	100	25	9	183	57	7	118
*Bothropoides jararaca*	500	24	8	193	61	9	160
*Bothropoides lutzi*	400	23	9	170	42	6	80
*Bothropoides mattogrossensis*	250	24	9	175	49	7	130
*Bothropoides neuwiedi*	500	26	9	172	45	9	100
*Bothropoides otavioi* e	100	24	8	187	63	5	74.4
*Bothropoides pauloensis*	400	24	9	174	47	8	93.8
*Bothropoides pubescens*	250	25	9	176	45	8	120
*Bothrops asper*	1,320	27	7	201	64	8	250
*Bothrops atrox*	640	24	7	192	67	7	172.3
*Bothrops barnetti*	0	24	8	178	44	7	140
*Bothrops brazili*	250	26	8	177	55	7	149.3
*Bothrops caribbaeus*	100	27	7	205	68	5	200
*Bothrops jararacussu*	350	25	8	176	56	6	220
*Bothrops lanceolatus*	650	31	8	224	64	8	200
*Bothrops leucurus*	200	27	8	208	66	8	120
*Bothrops lojanus*	2,200	23	8	150	42	5	61
*Bothrops marajoensis*	50	25	8	185	59	7	150
*Bothrops moojeni*	750	26	7	195	57	10	230
*Bothrops muriciensis*	640	25	8	153	52	6	88.4
*Bothrops osbornei*	1,250	26	8	179	67	7	140
*Bothrops pirajai*	250	26	9	161	48	6	137
*Bothrops punctatus*	1,607	27	8	204	83	8	130
*Bothrops roedingeri*	250	21	11	176	43	6	100
*Bothrops sanctaecrucis*		25	8	181	57	8	100
*Bothrops venezuelensis*	1,500	24	8	199	61	6	166.7
*Cerrophidion godmani* f	2,005	21	9	141	29	5	82.2
*Cerrophidion petlalcalensis*	2,200	19	10	140	36	3	50
*Cerrophidion sasai*	1,990	21	9	139	30	3	71.3
*Cerrophidion tzotzilorum*	2,275	21	10	128	27	3	50
*Cerrophidion wilsoni* d	2,356	21	9	142	30	5	78.9
*Crotalus adamanteus*	250	29	15	176	27	8	251.1
*Crotalus aquilus*	2,355	23	12	148	25	3	67.8
*Crotalus atrox*	750	25	15	182	24	5	234
*Crotalus basiliscus*	1,200	27	15	192	27	2	204.5
*Crotalus catalinensis*	235	25	15	183	23	5	73.1
*Crotalus cerastes*	600	21	13	143	20	5	82.4
*Crotalus durissus*	500	27	15	173	25	2	180
*Crotalus enyo*	0	25	14	169	25	5	89.9
*Crotalus ericsmithi* g	1,037	25	13	172	41	5	54
*Crotalus horridus*	1,000	23	14	171	23	6	189.2
*Crotalus intermedius*	2,500	21	9	168	24	3	57
*Crotalus lannomi* h	978	27	15	172	42	4	63.8
*Crotalus lepidus*	1,650	23	13	160	25	2	82.8
*Crotalus mitchellii*	1,220	25	16	173	22	5	136.7
*Crotalus molossus*	1,465	27	17	182	23	3	133
*Crotalus oreganus*	1,250	25	15	179	22	5	162.6
*Crotalus polystictus*	2,025	27	14	174	23	3	100
*Crotalus pricei*	2,527	21	9	154	26	3	66
*Crotalus pusillus*	1,953	23	12	156	29	3	68.2
*Crotalus ravus*	2,245	22	11	143	25	1	70
*Crotalus ruber*	750	29	17	193	22	7	162
*Crotalus scutulatus*	900	25	15	179	22	2	137.3
*Crotalus simus*	500	29	14	181	26	4	180
*Crotalus stejnegeri*	860	27	15	175	41	7	72.4
*Crotalus tancitarensis* i	2,375	21	9	159	22	2	41
*Crotalus tigris*	733	23	13	167	22	6	88.5
*Crotalus tortugensis*	105	27	16	185	21	4	105.8
*Crotalus totonacus*	840	25	14	190	26	2	166.5
*Crotalus transversus*	3,250	21	9	146	23	3	46.5
*Crotalus triseriatus*	3,536	23	12	140	26	3	68.3
*Crotalus viridis*	1,438	27	14	179	23	5	151.5
*Crotalus willardi*	2,205	25	14	153	29	8	67
*Lachesis acrochorda*	250	35	9	220	43	13	300
*Lachesis melanocephala*	500	37	8	216	45	12	240
*Lachesis muta*	500	35	9	225	44	11	360
*Lachesis stenophrys*	500	35	8	200	43	12	348.7
*Mixcoatlus barbouri* j	2,217	17	9	140	30	4	51.2
*Mixcoatlus browni* j	2,563	19	8	139	30	1	51.5
*Mixcoatlus melanurus*	2,000	21	11	147	40	11	57.5
*Ophryacus undulatus*	2,300	21	12	168	47	15	70
*Porthidium arcosae*	150	27	9	166	33	5	63.5
*Porthidium dunni*	350	23	10	148	37	5	54.2
*Porthidium hespere*	300	23	10	156	30	5	57.9
*Porthidium lansbergii*	635	24	9	153	34	6	90
*Porthidium nasutum*	450	23	10	134	32	5	63.5
*Porthidium ophryomegas*	500	25	10	166	38	6	77
*Porthidium porrasi*	100	25	10	138	29	6	70
*Porthidium volcanicum*	500	25	10	161	30	7	53.6
*Porthidium yucatanicum*	125	25	10	148	37	5	59.8
*Rhinocerophis alternatus*	350	30	9	173	42	11	169
*Rhinocerophis ammodytoides*	1,000	24	10	154	36	9	75
*Rhinocerophis cotiara*	900	27	10	162	40	12	94.5
*Rhinocerophis fonsecai*	1,300	27	9	172	48	9	107.9
*Rhinocerophis itapetiningae*	750	26	8	152	32	7	50
*Rhinocerophis jonathani* k	3,010	28	11	162	38	11	88.1
*Sistrurus catenatus*	1,513	25	12	145	28	1	100.3
*Sistrurus miliarius*	250	23	11	135	32	1	80.3

Note

All literature derived from Campbell & Lamar, 2004 except where noted.

Abbreviations: Elev, elevation; ISO, intersupraoculars; MDSR, mid‐dorsal scale rows; SC, subcaudal scales; SL, supralabial scales; TL, total length; Ven, ventral scales.

a McCranie, Orellana, and Sheehy (2013).

b Jadin et al. (2010).

c Townsend, Medina‐Flores, Wilson, Jadin, and Austin (2013).

d McCranie (2011).

e Barbo, Grazziotin, Sazima, Martins, and Sawaya (2012).

f Jadin et al. (2012).

g Campbell and Flores‐Villela (2008).

h Reyes‐Velasco, Grünwald, Jones, and Weatherman (2010).

i Alvarado‐Díaz and Campbell (2004).

j Jadin et al. (2011).

k Carrasco, Harvey, and Saravia (2009).

### 2.4 Statistical analyses

To test for a relationship between maximum body size and elevation, we used a general linear mixed effects model in R with the package “nlme” (Pinheiro, Bates, DebRoy, Sarkar, & R Core Team, 2014; R Core Team, 2013). We included an initial random intercept and slope structure of genus nested within clade to account for nonindependence of each observation due to species relatedness. In other words, the model accounts for the fact that the body sizes of snakes within a genus (and clade) are likely more similar to each other than to a random species outside of this genus (or clade). We then used likelihood ratio testing to identify the best random structure for the model. We chose this nested random effect method of accounting for nonindependence among snake species instead of using a linear model with phylogenetically independent contrasts because we did not have a fully resolved molecular phylogeny for the entire group of snakes. For the analysis, we log_10_‐transformed maximum body size and square‐root transformed midpoint elevation in order to meet the assumptions of normally distributed residuals, and we therefore used a Gaussian family error distribution with identity link. We also conducted separate regression analyses for the three most speciose clades: the *Porthidium* group, the rattlesnakes, and the South American lanceheads.

**Figure 2:**
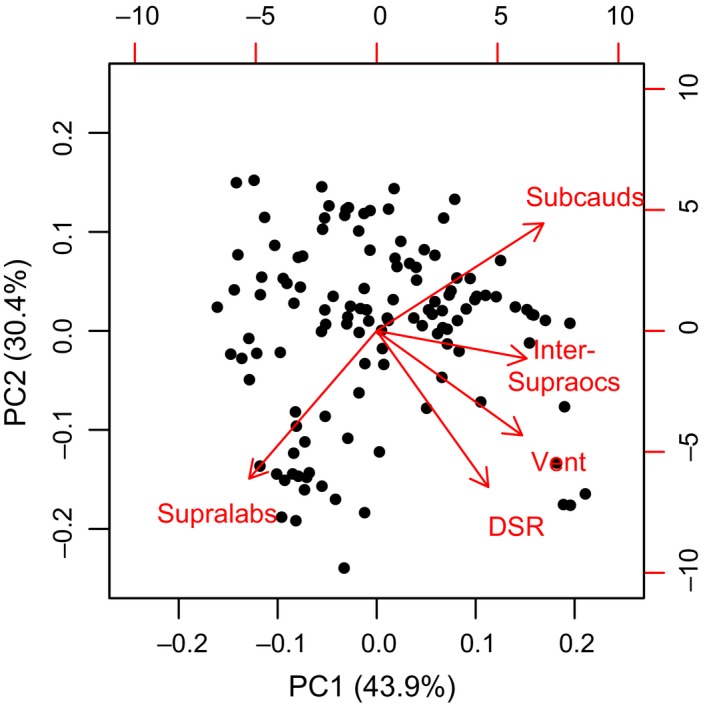
Biplot displaying the relationship of scale counts to the two primary axes of the principle components analysis (PCA). The percentages on the axis labels represent the amount of variation in the scale data explained by each axis, cumulating to over 74% of the total variation. Vector directions represent the rotations of each variable on the axes, and vector lengths represent how well each variable is represented by each axis

We then determined how scale counts were related to body size and elevation. First, in order to collapse the highly correlated data on scale counts, we performed a principal components analysis (PCA) on the log‐transformed (centered and scaled) scale count data. The first axis of the PCA explained 44% of the variation in the data set, and the loadings from this axis were used as a proxy for overall scale counts. This first axis was positively correlated with all scale characteristics except the modal supralabial counts, allowing us to easily interpret higher PCA scores as representing generally higher scale counts (Figure 2). To statistically model the effects of elevation and body size on scale counts, we again used a general linear mixed effects model with the same initial random effects structure and data transformations as above. We also calculated the variance inflation factor (VIF = 1.12), which verified that the colinearity between elevation and maximum body size was not strong enough to bias the model (Fox, 2008).

## 3 RESULTS

**Figure 3:**
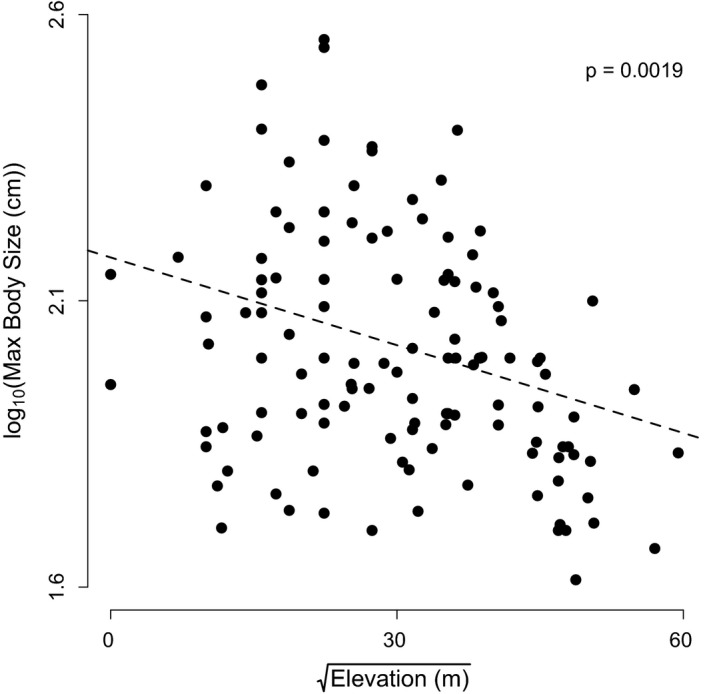
The relationship between maximum body size and elevation. Note that the line of best fit incorporates the random effect of genus within clade. Thus, the line represents the effect of elevation for the “average” genus

**Figure 4:**
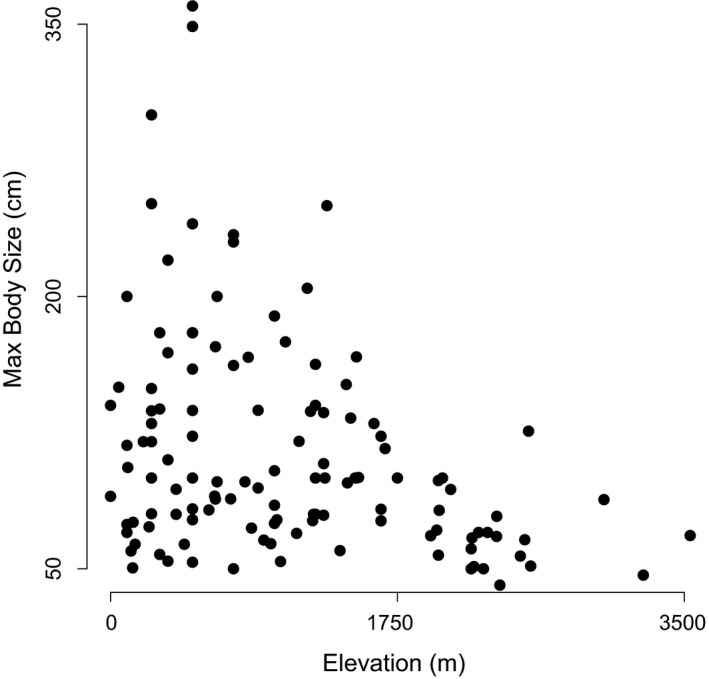
The relationship between maximum body size and elevation without transformation

**Figure 5:**
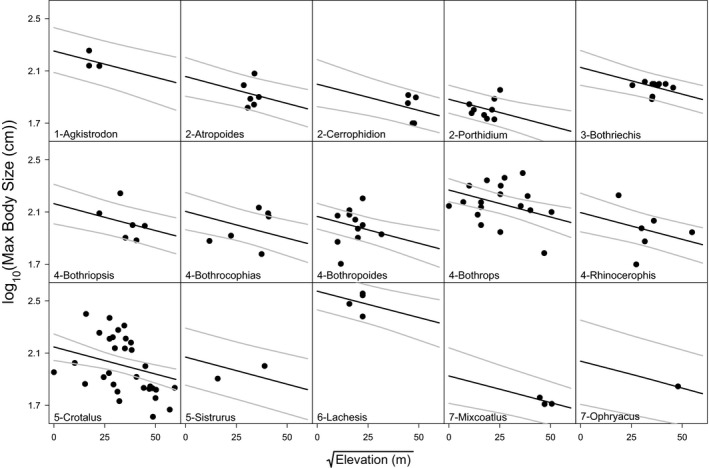
The relationship between maximum body size and elevation for each genus. Unique intercepts result from the significant random effect on the intercept, where genus is nested within clade. The gray lines represent the 95% confidence prediction intervals taking into account the uncertainty in the intercepts and the overall slope. Clades are represented by the number preceding the genus labels

We found that, when we look across all clades, maximum body size significantly declines with elevation in pitvipers, displaying a contra‐Bergmann's rule pattern (overall effect of elevation: *t*_106_ = −3.19, *p* = .0019; Figures 3, 4, 5). The best random structure for this model included the nested random intercepts of both clade and genus, but did not include a random effect on the slopes. In order to describe how well our model explained the relationship between body size and elevation, we calculated the marginal and conditional *R* ^2^ values for the model (Johnson, 2014; Nakagawa & Schielzeth, 2013), where the marginal *R* ^2^ (0.04) describes the proportion of variance explained by elevation alone, and the conditional *R* ^2^ (0.64) describes the proportion of variance explained by both elevation and the random effects of genus and clade. This implies that the negative relationship between body size and elevation was strongly driven by the differences in body size among clades. Specifically, genera with larger bodied species (e.g., *Lachesis*) tend to occupy lower elevations, while clades with smaller bodied species tend to occupy high elevations (e.g., *Cerrophidion* and *Mixcoatlus*; Figure 5). Still, when we analyzed the three most speciose clades separately, rattlesnakes in the genus *Crotalus* showed an independently significant negative relationship between body size and elevation (*t*_34_ = −2.84, *p* = .0077), although the other two clades did not (*Porthidium* group: *t*_20_ = 0.52, *p* = .61; South American lanceheads: *t*_41_ = −0.72, *p* = .48). Therefore, our analyses suggest that the negative pattern between body size and elevation can be seen both among and within pitviper clades, although the pattern within clades is generally weaker.

**Figure 6:**
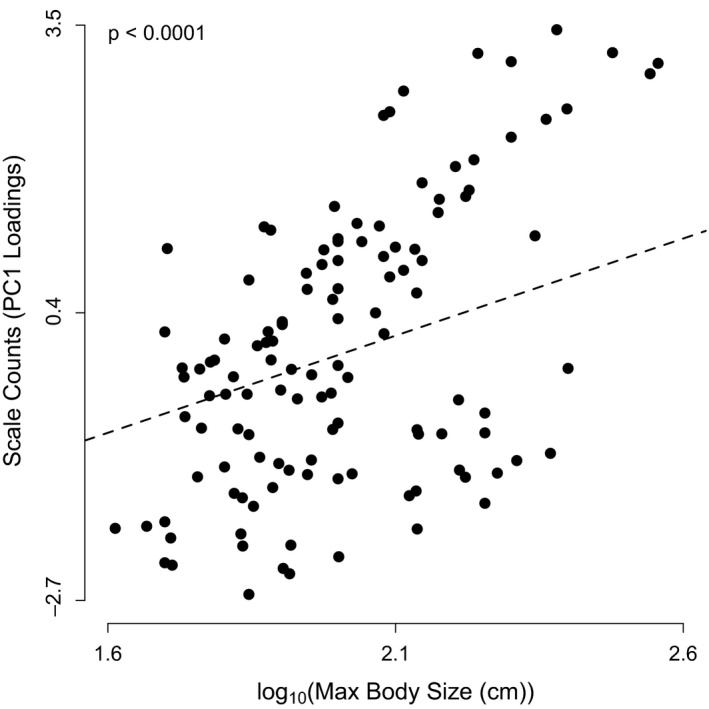
The overall relationship between scale counts and maximum body size. Note that the line of best fit incorporates the random effect of genus within clade. Thus the line represents the effect of body size for the “average” genus

**Figure 7:**
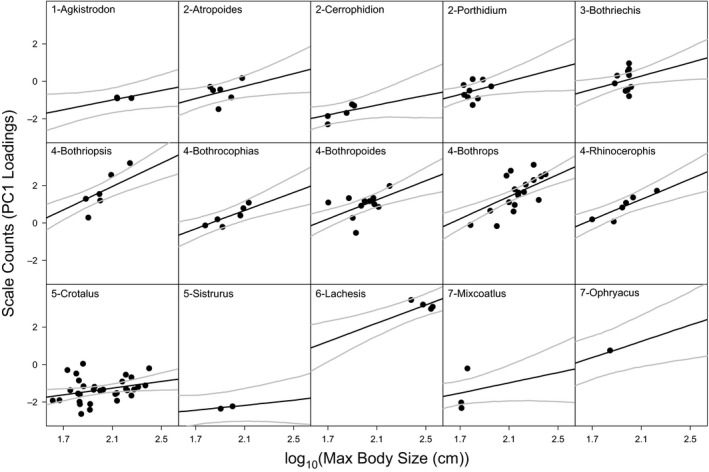
The relationship between scale counts and maximum body size. Unique intercepts and slopes result from the random effects, where genus is nested within clade. However, it should be noted that, for this model, there was no clear difference between a model with no random slope. The gray lines represent the 95% confidence prediction intervals taking into account the uncertainty in the intercepts and the overall slope. Clades are represented by the number preceding the genus labels

We found that scale counts (PC1 loadings) significantly increased with increasing body size, but there was no significant effect of elevation (*t*_106_ = 5.49, *p* < .0001; marginal *R* ^2^ = .08, conditional *R* ^2^ = .83; Figure 2), again with a strong effect of the differences among clades. PC1 loadings positively correlate with the counts of nearly all scale types, suggesting that scale counts generally increase with body size. This positive association was clear both among and within clades and genera (Figures 6 and 7). In this case, the model that included random slopes and intercepts of clade and genus was indistinguishable from one that only included random intercepts. This suggests that the relationship (i.e., slope) between scale count and body size is similar among all pitvipers, though slight differences may exist among and within clades (Figure 7).

## 4 DISCUSSION

Across 15 genera of New World pitvipers, the data support a negative relationship between elevation and body size and a positive relationship between scale counts and body size (Figures 3 and 6), although elevation does not seem to directly correlate with scale counts after accounting for body size. The negative relationship between body size and elevation was clearly supported across taxonomic levels. When we examined patterns among genera, we observed larger bodied clades (e.g., *Lachesis*) at low elevations and smaller bodied clades (e.g., *Mixcoatlus*) at high elevations. Among the more speciose clades examined separately, only the genus *Crotalus*, which spans the largest range of elevation, was found to have a significant negative relationship between body size and elevation (Figure 5). Therefore, our results corroborate Cruz et al. (2005) that the taxonomic scale of analysis and geographic distributions of particular groups may likely influence the ability to detect Bergmann's rule within and among taxa.

Research investigating how body size changes along environmental gradients in squamate reptiles has found no consistent pattern (Oufiero et al., 2011). Some studies identified negative correlations between body size and latitude and elevation (Ashton & Feldman, 2003), while others have found positive relationships with latitude and no relationships with elevational range (Cruz et al., 2005). Within some studies, effects of latitude were stronger when elevation was taken into account (Cruz et al., 2005; Olalla‐Tárraga et al., 2006; Terribile et al., 2009). Our study contributes to this growing body of research, specifically for elevational gradients, by supporting a contra‐Bergmann's pattern across 15 genera in the New World pitvipers. Our results are similar to those found by Reed (2003) but in direct contrast to findings of Olalla‐Tárraga et al. (2006) where the largest species of snakes occurred at higher, colder elevations in Western North America. Different patterns across studies could also be based on the geographic ranges of species studied. For example, Terribile et al. (2009) found that vipers follow a contra‐Bergmann's pattern when analyses were restricted to South America but not North America. Similarly, in Europe snakes increased in body size with decreasing latitude, but the pattern was inconsistent and more complex in North America (Olalla‐Tárraga et al., 2006). Additional discrepancies among studies may be related to different phylogenetic levels displaying significant variation in patterns of body size (Cruz et al., 2005; Olalla‐Tárraga et al., 2006; Terribile et al., 2009). For example, eastern clades of *Crotalus viridis *sensu* lato* followed Bergmann's rule, while western clades showed the opposite trend (Ashton, 2001; Cruz et al., 2005). Different patterns of body size distributions across clades may also be the result of different physiological mechanisms (Terribile et al., 2009). Some of the most common mechanisms hypothesized to contribute to Bergmann's rule include heat conservation (Ashton & Feldman, 2003; Cowles, 1945; Olalla‐Tárraga et al., 2006), embryonic development (Angilleta, Niewiarowski, Dunham, Leaché, & Porter, 2004; Oufiero et al., 2011; Tousignant & Crews, 1995), prey availability (Ashton & Feldman, 2003), and sexual selection (Pincheira‐Donoso et al., 2008). However, our analyses were focused on correlations and could not distinguish among these hypotheses, making this an important subject of further investigation.

Physiological mechanisms involving temperature may also contribute to variation in other physical features and display variation along elevational gradients (Bergmann, 1847; Cowles, 1945; Watt et al., 2010). As an important first step, we described the pattern of scale counts with respect to both body size and elevation because of the potential role scales play in physiological processes of reptiles. For example, small *Sceloporus* occurring at high elevations have numerous small scales, while larger bodied, low elevation species have fewer larger scales (Bogert, 1949; Oufiero et al., 2011). It is hypothesized that, for lizards, having smaller, more numerous scales in colder environments facilitates heat retention, while the larger scales seen in warmer climates function as heat shields (Oufiero et al., 2011; Regal, 1975). Additionally, squamate skin influences cutaneous evaporation with large scales potentially associated with higher rates of water loss (Martínez‐Feíra, Santos, Pleguezuelos, Lizana, & Brito, 2009; Oufiero et al., 2011; Sanders et al., 2004; Soulé & Kerfoot, 1972). This is supported by patterns of *Sceloporus* with fewer scale rows found with decreasing aridity (Oufiero et al., 2011) and higher numbers of ventral scales in snakes from drier, open habitats (Martínez‐Feíra et al., 2009). Generally, our findings of increased scale count at low elevations in pitvipers contrast to these previous studies on lizards. However, our finding may purely result from larger pitvipers, with more scales, being found at lower elevations and vice versa.

Our observed trends suggest that temperature related mechanisms may have different effects between lizards and snakes or that other climatic or habitat variables have a significant influence on scale characteristics in snakes. For example, squamate skin influences cutaneous evaporation with large scales potentially associated with higher rates of water loss (Martínez‐Feíra et al., 2009; Oufiero et al., 2011; Sanders et al., 2004; Soulé & Kerfoot, 1972). This is supported by patterns of *Sceloporus* with fewer scale rows found with decreasing aridity (Oufiero et al., 2011) and higher numbers of ventral scales in snakes from drier, open habitats (Martínez‐Feíra et al., 2009). Another key aspect to scales related to variation in habitat is locomotor performance (Kelley, Arnold, & Gladstone, 1997). Snakes may have a closer functional association between scale counts and locomotor ability due to the elongation of the body and loss of limbs (Kerfoot, 1970). Furthermore, morphological traits required for specific habitats may be associated with different patterns of scale counts. For example, arboreality results in more elongated bodies that may have a corresponding influence on ventral scale numbers such as in *Bothriopsis taeniata* (Martins, Araujo, Sawaya, & Nunes, 2001; Terribile et al., 2009). One example species that appears to deviate from our general scale trends is the Sidewinder Rattlesnake, *Crotalus cerastes*, which is a species occurring at low elevations but has a small body and numerous squamations. These scale number adaptations may be a result of movement and survival constraints in hot, dry, and sandy deserts. Therefore, we believe that species diverging from Bergmann's or contra‐Bergmann's rules likely have specialized adaptations to ecological habitats (e.g., arboreal, semiaquatic) with stronger natural selective pressure on scale counts and body size than climate variation associated strictly with elevation alone.

Our results suggest important potential mechanisms for future investigation but should be interpreted in light of the limitations of data collection and study design. First, our approach limits environmental gradients to a single point in geographical or environmental space and ignores the interaction of these factors (Blackburn & Ruggiero, 2001; Gaston et al., 2008; Olalla‐Tárraga et al., 2010; Ruggiero & Lawton, 1998). In addition, although elevation as a proxy is extremely common in this literature, this is an assumption that might not show strict associations in all cases (Oufiero et al., 2011). We also did not include any information about latitude in our analysis, which further affects climate. Future research could investigate the patterns of pitviper body size and scale traits with climatic factors directly, such as georeferenced snake specimens that can be directly linked to a local climate. Second, by analyzing single data points for species level and above, we cannot address how population‐level variation in both body size and scale numbers influences the associations with elevation or climate (Martínez‐Feíra et al., 2009). Third, our general approach is based on availability of data from the literature, and some sample sizes from which the values were taken may not accurately represent the taxa studied (Gaston et al., 2008). Finally, the marginal *R* ^2^ value was low (.04) for the relationship between elevation and body size, suggesting that there are additional factors driving body size distributions beyond elevational ranges.

Overall, our results contribute to the generality of documented patterns of body size clines in reptiles in the western hemisphere and our understanding of evolutionary and ecological mechanisms shaping reptile species richness, distribution, and assemblages (Adams & Church, 2008; Ashton & Feldman, 2003; Cruz et al., 2005; Gaston et al., 2008; Olalla‐Tárraga et al., 2006). By examining scale traits in addition to body size, our study also has important implications for taxonomy. Among widely distributed taxa over diverse habitats, confusion of taxa may exist because most species descriptions depend on morphological traits (Sanders et al., 2004). For example, similar selective pressures can result in similar morphological traits of closely related species of viper (Martínez‐Feíra et al., 2009). Our research also has implications for understanding the role of climate change on pitvipers due to potential shifts in elevational ranges and decreasing body size (Cruz et al., 2005; O'Brien, Fox, Planque, & Casey, 2000; Portner, 2001). Better understanding of the relationship between climate and morphology may support the development of new hypotheses pertaining to the response of these taxa to climate change and enhance conservation efforts (Oufiero et al., 2011; Sinervo et al., 2009).

## CONFLICT OF INTEREST

None declared.

## AUTHOR CONTRIBUTIONS

RCJ conceived the study, while SAO and JRM improved on those ideas; RCJ collected the data; JRM, SAO, and RCJ analyzed the data; All authors contributed to the writing of the paper and have approved of the final version.
